# An engineered *Mycoplasma pneumoniae* to fight *Staphylococcus aureus*

**DOI:** 10.15252/msb.202110574

**Published:** 2021-10-06

**Authors:** Dominick Matteau, Sébastien Rodrigue

**Affiliations:** ^1^ Département de Biologie Université de Sherbrooke Sherbrooke QC Canada

**Keywords:** Biotechnology & Synthetic Biology, Microbiology, Virology & Host Pathogen Interaction

## Abstract

Bacterial infections are commonly treated with antimicrobials, but the rise of multi‐drug resistance and the presence of biofilms can compromise treatment efficacy. Recently, new approaches using live bacteria or engineered microorganisms have gained attention in the fight against several diseases. In their recent work, Lluch‐Senar and colleagues (Garrido *et al*, 2021) genetically modified the lung pathogen *Mycoplasma pneumoniae* to attenuate its virulence and secrete antibiofilm and bactericidal enzymes. Their strategy successfully altered a *Staphylococcus aureus* biofilm on catheters implanted in mice, providing an additional demonstration of the potential of genetically engineered microorganisms as therapeutic agents.

## Graphical abstract

Multi‐drug resistance and the presence of biofilms can compromise antimicrobial treatment efficacy. In their recent work, Lluch‐Senar and colleagues (Garrido *et al*, 2021) engineered the lung pathogen *M. pneumoniae* to secrete antibiofilm and bactericidal enzymes.

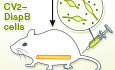

Biofilms are composed of microorganisms embedded within a complex extracellular matrix. This structure provides a physical barrier protecting microbes from desiccation and other stresses. For example, antibiotic susceptibility of bacterial populations contained within a biofilm is considerably decreased, often by up to 1,000‐fold (Høiby *et al*, 2010). Biofilms are commonly found on medical devices such as catheters and are notoriously difficult to eliminate, which can result in infections. Given the lack of efficient solutions to dissolve biofilms, combined with the steady rise in antibiotic resistance worldwide, new strategies must be developed to maintain the ability to prevent or treat infections.

During the past decades, most drugs consisted of molecules displaying pharmacological activities. Different levels of drug complexity are possible ranging, for example, from simple antibiotic molecules such as penicillin to proteins such as insulin or monoclonal antibodies. But what if we could use live organisms as drugs? Bacteriophages can be used to treat infections (Fathima & Archer, 2021), and probiotics are already well known to the general public. These natural organisms can have beneficial effects, but their impact on health or disease often remains limited. Could we instead rationally design microorganisms to prevent or treat specific diseases? This idea is particularly attractive given the remarkable progress of synthetic biology. In principle, engineered organisms also offer countless possibilities.

Medicines composed of living cells are generally referred to as live biotherapeutics products (LBPs) (Pot & Vandenplas, 2021). Several academic and industrial laboratories worldwide are engineering LBPs. The cell chassis usually consists of well‐established laboratory workhorses such as *Escherichia coli* or *Lactococcus* sp. (Aggarwal *et al*, 2020), largely due to the wealth of data and robust methods developed for these bacterial species. However, many less conventional organisms could be better suited for certain indications or present other advantages. Surprisingly, few people are aware that the lung pathogen *Mycoplasma pneumoniae* is one of the best‐studied microorganisms. For the past 15 years, an impressive amount of work was performed on this wall‐less bacterium, resulting in a detailed cellular description (Güell *et al*, 2009; Kühner *et al*, 2009; Yus *et al*, 2009; Trussart *et al*, 2017). More recently, the genetic engineering toolbox available for *M*. *pneumoniae* was expanded to considerably facilitate genome modifications, enabling a new level of studies with this near‐minimal bacterium (Piñero‐Lambea *et al*, 2020).

In their recent study, Lluch‐Senar and colleagues (Garrido *et al*, 2021) performed the first engineering and evaluation of *M. pneumoniae* as an LBP chassis (Fig 1). They first investigated the impact of selected gene deletions to attenuate this pathogen based on data from the literature, and tested the resulting strains using a mouse mastitis model of infection. Two genes, *mpn133* and *mpn372*, were removed to obtain a fully attenuated cell chassis called CV2. The team next identified signal peptides and assessed different promoters to allow efficient protein secretion. The signal peptide from gene *mpn142* was found to provide the best results and was further optimized to remove secondary structures from its mRNA counterpart. A genetic module allowing the secretion of dispersin B was then introduced in the CV2 strain to create an LBP capable of degrading *Staphylococcus aureus* biofilms. Surprisingly, this derivative of the CV2 strain was clearly surpassed by the wild‐type *M*. *pneumoniae* secreting dispersin B *in vivo* but not *in vitro* or *ex vivo*. This result suggested that the attenuation of the strain impaired its activity in mice and that additional factors such as inflammation could be important to eliminate *S*. *aureus* biofilms *in vivo*. Faced with this situation, the authors inserted another genetic module, this time expressing the glycylglycine endopeptidase lysostaphin that attacks the *S. aureus* cell wall. The CV2 strain secreting dispersin B and lysostaphin showed improved activity although with more variability than the wild‐type strain producing dispersin B.

**Figure 1:**
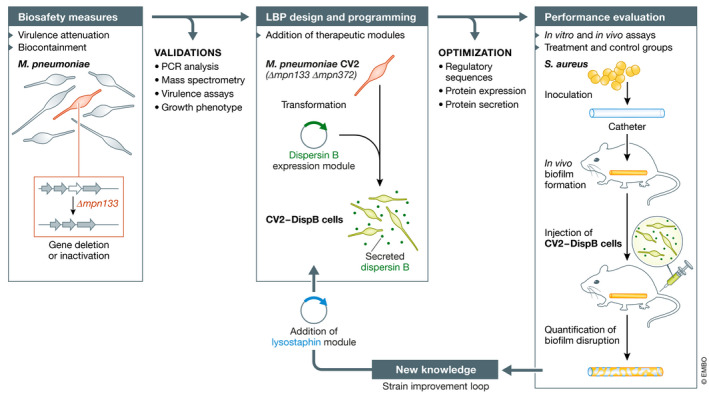
Main steps typically involved in the development of live biotherapeutics products (LBPs) Once a cell chassis is selected, the first step in LBP development generally consists in performing one or many genetic modifications to ensure the biosafety of the product for its intended usages (left panel). These modifications can, for example, aim at attenuating the virulence of the selected organism or at adding biocontainment measures such as nutritional dependencies. Different validations can be performed to confirm the desired phenotype. In the case of *M*. *pneumoniae*, deleting both *mpn133* and *mpn372* genes (CV2 mutant) resulted in a significant decrease in virulence as shown using a mouse mammary gland infection model, in which the pro‐inflammatory response was also significantly lowered compared with the wild‐type strain (Garrido *et al*, 2021). The second step of LBP creation involves the addition or programming of therapeutic modules and optimization efforts for cells to properly execute the designed genetic program (middle panel). For example, Lluch‐Senar and colleagues (Garrido *et al*, 2021) complemented *M. pneumoniae* with a genetic module allowing the secretion of dispersin B, a glycosyl hydrolase capable of dissolving *S. aureus* biofilms. With the therapeutic modules loaded into the selected organism, the final step consists of different experiments to evaluate the overall performance of the LBPs (right panel). These experiments usually begin with *in vitro* assays, followed by more complex investigations using *in vivo* models. To demonstrate the therapeutic potential of *M. pneumoniae* secreting dispersin B (CV2‐DispB), Lluch‐Senar and colleagues notably used a mouse model in which catheters containing *in vivo*‐developed biofilms were treated with subcutaneous injections of *M. pneumoniae* CV2‐DispB. Following the first round of LBP development, new knowledge emerges and the cell chassis can be further improved, as demonstrated by the addition of the lysostaphin module into *M. pneumoniae* CV2‐DispB to increase its activity against *S. aureus* biofilms.

Overall, the work conducted by Lluch‐Senar and colleagues is promising. Importantly, several relevant conclusions can be drawn from their experiments. First, alternative model organisms, such as *M. pneumoniae*, deserve to be considered in LBP design. The lack of a cell wall in *M. pneumoniae* facilitates the secretion of bioactive compounds and the production of molecules targeting bacterial peptidoglycans. Second, knowledge is key for rational engineering. Having access to solid data from the literature and validating hypotheses *in vivo* is crucial. Surprises related to the engineered organism behavior are likely, particularly if some initial information used for the design comes from different sources or contexts. For instance, virulence genes in *M. pneumoniae* were identified for lung infection, while the CV2 strain was evaluated in a mouse mastitis model and finally used to degrade biofilms on catheters inserted subcutaneously. Third, reassessment and iterative strain improvement will likely be needed to reach the desired efficacy. Finally, strict biocontainment measures will certainly be required by regulatory agencies and for the acceptance of LBPs by the public. Minimizing the risk of infection in other organs and avoiding dissemination to other patients or in the environment is an obvious condition for clinical use.

With the advances in synthetic biology and in the engineering of LBPs, several applications are expected to be developed in the coming years. We are only witnessing the beginning of this exciting field that has the potential to prevent or treat a wide variety of diseases such as multi‐drug‐resistant infections and even cancer. LBPs could provide new therapeutic avenues where conventional drugs have failed or reached their limits.
